# GNNGL-PPI: multi-category prediction of protein-protein interactions using graph neural networks based on global graphs and local subgraphs

**DOI:** 10.1186/s12864-024-10299-x

**Published:** 2024-05-09

**Authors:** Xin Zeng, Fan-Fang Meng, Meng-Liang Wen, Shu-Juan Li, Yi Li

**Affiliations:** 1 College of Mathematics and Computer Science, Dali University, 671003 Dali, China; 2 State Key Laboratory for Conservation and Utilization of Bio-Resources in Yunnan, Yunnan University, 650000 Kunming, China; 3 Yunnan Institute of Endemic Diseases Control & Prevention, 671000 Dali, China

**Keywords:** Multi-category prediction of protein-protein interactions, Graph neural network, Global graphs, Local subgraphs, Asymmetric loss function

## Abstract

Most proteins exert their functions by interacting with other proteins, making the identification of protein-protein interactions (PPI) crucial for understanding biological activities, pathological mechanisms, and clinical therapies. Developing effective and reliable computational methods for predicting PPI can significantly reduce the time-consuming and labor-intensive associated traditional biological experiments. However, accurately identifying the specific categories of protein-protein interactions and improving the prediction accuracy of the computational methods remain dual challenges. To tackle these challenges, we proposed a novel graph neural network method called GNNGL-PPI for multi-category prediction of PPI based on global graphs and local subgraphs. GNNGL-PPI consisted of two main components: using Graph Isomorphism Network (GIN) to extract global graph features from PPI network graph, and employing GIN As Kernel (GIN-AK) to extract local subgraph features from the subgraphs of protein vertices. Additionally, considering the imbalanced distribution of samples in each category within the benchmark datasets, we introduced an Asymmetric Loss (ASL) function to further enhance the predictive performance of the method. Through evaluations on six benchmark test sets formed by three different dataset partitioning algorithms (Random, BFS, DFS), GNNGL-PPI outperformed the state-of-the-art multi-category prediction methods of PPI, as measured by the comprehensive performance evaluation metric F1-measure. Furthermore, interpretability analysis confirmed the effectiveness of GNNGL-PPI as a reliable multi-category prediction method for predicting protein-protein interactions.

## Introduction

Protein-protein interactions (PPI) play a crucial role in various biological processes within cells. Identifying PPI is of great significance in advancing research across multiple life science fields, including medical diagnosis, drug design, and disease treatment [1]. Currently, the methods for identifying PPI can be broadly categorized into traditional biological experimental methods and computational methods. Traditional biological experimental methods primarily involve techniques such as yeast two-hybrid [2], protein chips [3], and synthetic lethal analysis [4]. However, these methods suffer from several disadvantages, including being time-consuming, labor-intensive, and requiring high financial resources [5]. To overcome these disadvantages, the computational methods for PPI prediction have developed rapidly. Nevertheless, these computational methods face dual challenges. Firstly, they need to accurately identify multiple specific categories of PPI. Secondly, they must achieve the desired predictive performance.

In recent years, the computational methods for predicting PPI have transitioned from docking-based methods to machine learning and deep learning-based methods. Docking-based methods [6] are capable of effectively predicting PPI, but they require high-quality protein 3D structures and significant computational resources. Additionally, they operate at a slower prediction speed, which cannot keep pace with the demands of processing massive data. Machine learning and deep learning methods, on the other hand, have exhibited better performance in handling large amounts of data [7–10]. These methods can utilize both protein sequence and structural data. Machine learning-based methods [9, 11, 12] rely on protein sequence or structural features, utilizing models like SVM [10, 13, 14] and random forest [15] to predict PPI. Although these methods have displayed good predictive performance, they cannot automatically extract deep-level features of PPI from the original sequences or structures of proteins. This creates bottlenecks that hamper improvements in predictive performance. However, the emergence of deep learning models, such as multi-layer neural networks, has provided better model prediction performance and pointed researchers towards breakthroughs in addressing the performance bottlenecks encountered by machine learning techniques [16].

Methods for predicting PPI using deep learning techniques leverage protein sequences, structures, and PPI networks. Deep learning techniques such as deep neural networks (DNN) [17], convolutional neural networks (CNN) [18], recurrent neural networks (RNN) [19], attention mechanisms [20], and graph neural networks (GNN) [21] are employed to extract deep-level features from proteins and PPI networks. DNN-based methods extract protein features through multi-layer neural networks to directly predict PPI [22, 23] or employ machine learning models for PPI prediction [24]. CNN and RNN-based methods focus on extracting local features and long-range dependency features from protein sequences, respectively. For instance, LSTM-PHV method [8] and other related approaches [25] leveraged the LSTM (Long Short-Term Memory) model to capture long-range dependency features in protein sequences. Methods like ADH-PPI [26] and DCSE [27] integrated CNN and RNN to extract both local and long-range features from protein sequences, which were then combined to predict PPI. Attention mechanisms have also been widely utilized to identify key sequence features in protein sequences [23, 26, 28]. While these DNN, CNN, RNN, and attention-based methods primarily focused on protein sequence features, they often overlooked the structural features of proteins and the hidden interaction features present in PPI networks. To address these limitations, GNN-based methods have emerged. However, incorporating protein structures into these methods has been challenging due to the slow exploration of protein 3D structures. With the advent of protein 3D structure prediction tools like AlphaFold [29] and ColabFold [30], obtaining monomer protein 3D structures has become easier. This has led to rapid advancements in research that utilizes GNN to extract protein structural features for predicting PPI, either independently or in combination with protein sequence features. For instance, the method proposed in reference [31] directly employed the GCN/GAT model to extract the structural features from two interacting proteins. These features were then concatenated and used to predict PPI. TAGPPI method [32] combined TextCNN and GAT to extract sequence and structural features from protein sequences and contact maps, respectively. The extracted features were fused before being passed through a fully connected (FC) layer to predict PPI. Other methods introduced interaction features by constructing a PPI network graph where proteins served as vertices and interactions served as edges. GNN was then used to extract interaction features from this graph, ultimately predicting PPI. Methods such as S-VGAE [33], HIGH-PPI [34], and Topsy-Turvy [35] utilized PPI network graphs along with protein sequences or structures as vertex features. GNN was applied for binary classification to determine whether there was an interaction between vertices in the graph. The aforementioned methods treated PPI as a binary classification task and did not identify specific interaction categories between proteins. However, methods like PIPR [36], GNN-PPI [37], SemiGNN-PPI [38], and AFTGAN [39] have been developed to predict PPI into multi-category. PIPR utilized a Siemens residual current convolutional neural network (RCNN) to extract local features and contextual information from protein sequences. GNN-PPI, SemiGNN, and AFTGAN leveraged GNN to learn deep-level features from PPI networks, enabling multi-category prediction. Although these methods extracted protein sequence features and PPI network graph features for multi-category PPI prediction, research in this area was still in its early stages. The performance of the models was somewhat below user expectations. Therefore, proposing an efficient and effective model for multi-category prediction of PPI presents significant challenges.

In this study, we proposed a novel graph neural network method, called GNNGL-PPI, for predicting multi-category of protein-protein interactions. GNNGL-PPI utilized a combination of global graph and local subgraph features. Specifically, we used Graph Isomorphism Network (GIN) to extract global graph features from PPI network graphs. Additionally, we employed GIN-AK to extract local subgraph features from subgraphs containing protein vertices in the PPI network graphs. Simultaneously, to address the issue of imbalanced samples for each category in the benchmark dataset, we introduced an Asymmetric Loss (ASL) Function. This loss function helped enhance the predictive performance of the model by assigning different weights to different categories based on their prevalence. We evaluated the model on six standard test sets created using three different dataset partitioning algorithms (Random, BFS, DFS). GNNGL-PPI consistently outperformed the state-of-the-art methods for multi-category prediction of PPI, as measured by the comprehensive evaluation metric F1-measure. Furthermore, we conducted interpretability analysis to validate the effectiveness of GNNGL-PPI. Experimental results confirmed that GNNGL-PPI was a reliable method for predicting multi-category of PPI.

## Materials and methods

### Datasets

In this study, we approached protein-protein interactions prediction as a multi-category task, encompassing seven PPI categories: Reaction, Binding, Post-translational modification (Ptmod), Activation, Inhibition, Catalysis, and Expression. Each protein-protein interaction pair is assigned to at least one of these categories. For example, the interaction category between protein 9606.ENSP00000005257 (RalA) and protein 9606.ENSP00000202677 (RalGAP A2) is Inhibition (PMID: 34767674), while the interaction category protein 9606.ENSP00000003100 (Cyp51) and protein 9606.ENSP00000240055 (NF-YB) is Activation (PMID:27438,727). To evaluate the performance of the model, we utilized two benchmark datasets, SHS27k and SHS148k, which were consistent with the dataset used in the GNN-PPI [37] method and exhibited sequence consistency of less than 40% in each dataset. These datasets consisted of 7624 (1690 proteins) and 44,488 (5189 proteins) protein-protein interaction pairs, respectively. The number and occupation ratio of samples for the seven PPI categories generated by these protein-protein interaction pairs were shown in Table 1. It was evident from Table 1 that the number of samples for the seven PPI categories was imbalanced. During the training and testing of the model, we also employed the Random, Breath First Search (BFS), and Depth First Search (DFS) algorithms proposed by the GNN-PPI method to partition the SHS27k and SHS148k datasets. For detailed information on the data partitioning algorithms for the Random, BFS, and DFS, please refer to the GNN-PPI method.

**Table 1:** Number and occupation ratio of samples for seven categories of protein-protein interactions

PPI Categories	SHS27K		SHS148K	
	Number of Samples	Occupation Ratio	Number of Samples	Occupation Ratio
Reaction	3164	18.22%	18,067	17.71%
Binding	4017	23.13%	23,448	22.98%
Ptmod	1303	7.50%	9336	9.15%
Activation	3297	18.98%	18,910	18.53%
Inhibition	1407	8.10%	8987	8.81%
Catalysis	3492	20.11%	19,871	19.47%
Expression	687	3.96%	3419	3.35%

### PPI network graph formation and protein features

#### PPI network graph formation

Assuming a set of proteins P={p_1,p_2,…,p_n } with n as the number of proteins, each protein acted as a vertex in the PPI network graph. The interaction category between p_i and p_j was represented as edge e_ij (1≤i,j≤n). Different protein-protein interaction categories made up the category space D={D_1,…,D_t } (t=7) of the dataset, where t is the number of PPI categories. If there was an interaction between p_i and p_j of a certain category, the corresponding position in the adjacent matrix representing the PPI network graph was assigned a value of 1, otherwise, it was assigned a value of 0.

#### Protein features

We employed the pre-trained model MASSA [40] to capture high-level and more fine-grained features. This pre-trained model leveraged multi-modal protein data, including protein sequences, structures, gene ontology annotations, motifs, and region positions, to derive comprehensive protein features. Unlike previous research, which mainly focused on protein features derived from protein sequences such as Position-Specific Scoring Matrix (PSSM) [41] or Hidden Markov Models (HMM) [42] matrix, or treated protein sequences as natural language processing (NLP) [43] tasks to extract protein sequence features. However, these researches yielded relatively singular protein features and did not fully encompass other biochemical information pertaining to proteins.

### Proposed model

**Fig. 1:**
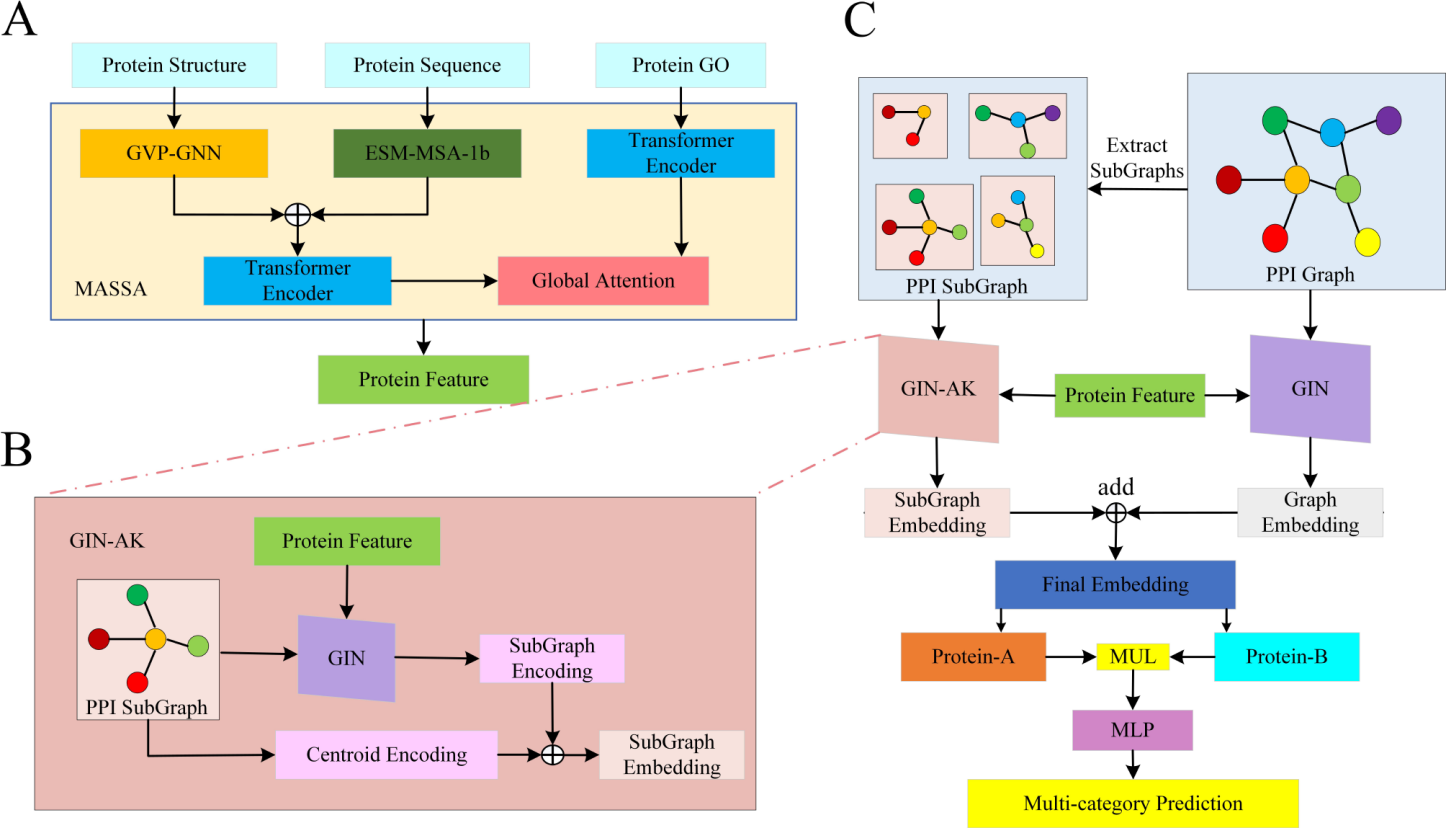
The architecture review of GNNGL-PPI. (**A**) We utilized the pre-training model MASSA to obtain the comprehensive protein features based on multimodal data such as protein structures, sequences, and gene ontology annotations. (**B**) GIN-AK extracted the global features and centroid features of subgraphs through two different processes, and then added these extracted features to obtain the final local subgraph features. (**C**) The extracted global features from the PPI network graph through GIN and the extracted local subgraph features by GIN-AK were concatenated to obtain the high-level features of vertices in the PPI network, i.e., protein features. We multiplied the features of proteins themselves and their interacting proteins and sent them into the multilayer perceptron (MLP) to complete multi-category prediction of protein-protein interactions

To begin with, we input the multimodal data of proteins, such as sequences, structures, and Go annotations, into the pre-trained model MASSA and obtained the 512-dimensional protein pre-training features. Next, we embedded the pre-trained features using a layer of linear transformation. Finally, we used the embedded features as the protein vertex features in the PPI network (Fig. 1A).

The extraction of high-level features in the PPI network graph can be divided into two main parts, as shown in Fig. 1B and C: global graph features extraction and local subgraph features extraction. In the global graph features extraction part, we utilized one layer of GIN followed by two FC layers. ReLU activation function [44] was applied in the FC layers. To ensure stable model training, batch normalization function was employed in the last layer to normalize the extracted features and obtained the global graph features of the PPI network graph. Moving on to the local subgraph features extraction section: firstly, we selected a protein vertex as the center and included all vertices within a path distance of K from that the center vertex. This formed a subgraph of the protein. Secondly, we used the GIN-AK to extract the features from the subgraph. GIN-AK incorporated two distinct processes to extract global features and centroid features of the subgraph, respectively. These features were then combined to obtain the final local subgraph features. To extract the global features of the subgraph, we input both the subgraph and protein features into a single layer of GIN. The output features of GIN were then fed into a FC layer (gating unit) with a sigmoid activation function, resulting in the global features of the subgraph. For the centroid features of the subgraph, we first calculated the path distance between the vertices in the subgraph. The obtained path distance matrix was then processed using the same gating unit to obtain the centroid features. Finally, we concatenated the global features and centroid features of the subgraph, applied batch normalization, and obtained the local subgraph features. The global graph features and local subgraph features were concatenated to form the vertex features in the PPI network graph. It was important to note that ReLU activation function was used in the gating unit during the subgraph features extraction process. Furthermore, to prevent gradient vanishing and enhance model stability during training, a dropout layer was added to the gating unit.

In order to predict multi-category of PPI, we first multiplied the features of the protein itself and its interacting protein. The resulting multiplied features were then fed into a FC layer. The output of the FC layer was a 7-dimensional matrix. Finally, by applying the sigmoid function to the 7-dimensional matrix, we completed the multi-category prediction of PPI.

### GIN-AK

GNN have proven to be an effective framework for topology representation learning. GIN is widely regarded as the most repressive GNN [45], but it still faces limitation in breaking through the first-order Weisfeiler-Leman isomorphism testing [46, 47]. To overcome this limitation, a subgraph operation had been introduced based on GIN. This approach updated vertex information by using the feature of the subgraph of vertex, rather than relying solely on the neighboring vertex feature information. This transformation reduced the complexity of the graph feature problem to a smaller and simpler subgraph feature problem. The resulting GIN model was called GIN-AK (GIN As Kernel). In the process of updating vertices using graph convolutional operations, each vertex aggregates information directly from neighboring vertices in a star operation. The star operation 

$Star\left(v\right)$ forms a graph that can be defined as formular (1). The specific update method for vertices in GIN is shown in formula 2.

1 $$ Star\left(v\right)=\left({\mathcal{N}}_{1}\left(v\right),\left\{(v,j)\in \epsilon|j\in \mathcal{N}(v)\right\}\right)$$

2 $$ {h}_{v}^{(l+1)}={GIN}^{\left(l\right)}\left({Star}^{\left(l\right)}\left(v\right)\right), {\forall }_{v}\in V,l=0,\dots,L-1$$

The limitation of graph convolutional operations lies in their inability to differentiate between graphs that possess the same vertex degree but exhibit different structures. In order to address this issue, we proposed the utilization of a subgraph encoder, denoted as $$ G\left[{\mathcal{N}}_{k}\left(v\right)\right]$$, to substitute the $$ Star\left(v\right)$$ operation. This replacement significantly enhanced the expressive capabilities of the graph convolutional operation. The subgraph encoder $$ G\left[{\mathcal{N}}_{k}\left(v\right)\right]$$ facilitated the update of vertex features by constructing subgraph features within a $$ k$$ -hop egonet centered around the vertex $$ v$$. This update process was displayed in formula (3), which specifically outlined the procedure for updating vertices in GIN-AK.

3 $$ {h}_{v}^{(l+1)}={GIN}^{\left(l\right)}\left({G}^{\left(l\right)}\left[{\mathcal{N}}_{k}\left(v\right)\right]\right), {\forall }_{v}\in V,,l=0,\dots,L-1$$

In GIN-AK, a $$ k$$ -hop propagation mechanism was employed to acquire the subgraph surrounding each vertex. Additionally, the path distance between each vertex and the centroid of its corresponding subgraph was computed. This information enhanced the vertex features and contributed to the overall improvement of graph convolutional expressiveness. To capture the nonlinear transformations of global features and centroid features of the subgraph, gating units were introduced. Finally, the global features and centroid features of the subgraph were obtained using formula (4) and (5), respectively.

4 $$ {h}_{v}^{\left(l+1\right)|subgraph}={Sigmoid\left({d}_{v|j}^{\left(l\right)}\right)\odot GIN}^{\left(l\right)}\left({G}^{\left(l\right)}\left[{\mathcal{N}}_{k}\left(v\right)\right]\right)$$

5 $$ {h}_{v}^{\left(l+1\right)|centroid}=Sigmoid\left({d}_{v|j}^{(l+1)}\right)\odot Emb\left(v\right|{G}^{\left(l+1\right)}\left[{\mathcal{N}}_{k}\left(v\right)\right])$$

Among them, $$ {d}_{v|j}^{(l+1)}$$represented the path distance matrix from vertex $$ v$$ to vertex $$ j$$ in the $$ l$$+1-layer, and $$ \odot $$ represented element wise multiplication. Combining the global features and centroid features of the subgraph, the update process of vertex $$ {h}_{v}^{(l+1)}$$ was shown in formula (6).

6 $$ {h}_{v}^{(l+1)}=SUM({h}_{v}^{\left(l+1\right)|subgraph},{h}_{v}^{\left(l+1\right)|centroid})$$

GIN-AK improved the expressiveness of graph convolutional operation through the use of subgraphs instead of star graphs. This enhancement allowed GIN-AK to capture underlying structural features in PPI network, leading to the improved performance in predicting different categories of protein-protein interactions.

### ASL

In this study, PPI prediction was regarded as a multi-category classification task. However, the dataset (Table 1) used was imbalanced, which means that there were more samples corresponding to some categories compared to others. This can affect the performance evaluation of the model since it might focus more on the majority classes and ignore the minority classes. To address this problem, we introduced the Asymmetric Loss (ASL) [48] function.

In multi-category imbalanced datasets, using symmetric loss functions like Focal Loss [49], BCE Loss [50], or Cross Entropy [51] may not effectively learn some features from positive samples. These loss functions tend to focus more on negative samples than positive samples [48], which can be suboptimal. For example, in Focal Loss function (formula 8), when using the same $$ \gamma $$ for multi-category training, it may eliminate the gradient of sparse positive samples. Among them, $$ p=\sigma \left(z\right)$$ is the output probability of the model, $$ \gamma $$ is the focusing parameter, $$ {L}_{+}$$ and $$ {L}_{-}$$ represent the positive and negative loss parts, respectively.

8 $$ \left\{\begin{array}{c}{L}_{+}={\left(1-p\right)}^{\gamma }log\left(p\right)\\ {L}_{-}={p}^{\gamma }\text{log}\left(1-p\right) \end{array}\right.$$

Therefore, in ASL, the focusing parameters of the loss function are decoupled into positive focusing parameter ${\gamma }_{+}$ and negative focusing parameter ${\gamma }_{-}$. This allows for asymmetric focusing, enabling better control the effect of positive and negative samples on the loss function. In addition, considering the asymmetric focusing parameters in ASL, there are shortcomings in some cases where there are not enough negative samples. In ASL, another asymmetric mechanism of probability translation is further proposed, as shown in formula (9).

9 $$ {p}_{m}=\text{m}\text{a}\text{x}\left(p-m,0\right)$$

$$ m$$ represents an adjustable probability margin. Through the above two adjustments, the final definition of ASL is shown in formula (10).

10 $$ ASL=\left\{\begin{array}{c}{L}_{+}={\left(1-p\right)}^{{\gamma }_{+}}log\left(p\right) \\ {L}_{-}={\left({p}_{m}\right)}^{{\gamma }_{-}}log\left(1-{p}_{m}\right)\end{array}\right.$$

By employing ASL to dynamically regulate the degree of asymmetry throughout the entire training process, the selection of hyperparameters is simplified. This effectively balances the focus of the network on positive and negative samples, ultimately improving the accuracy of multi-category prediction.

### Model training

We trained our model for 400 epochs using the Adam optimizer [52] with a batch size of 1024 and an initial learning rate of 0.01. To optimize the training process, we implemented a learning rate decay function, set the patience to 20, and stopped training when the model did not reduce its loss after 20 epochs. To prevent overfitting, we used a dropout rate of 0.2 in the local subgraph features extraction part and 0.5 in the global graph features extraction section. In the local subgraph features extraction part, we set $$ k$$ in $$ k$$-hop to 1, extracted the 1-hop subgraph as the input graph of GIN-AK, and used ASL with a probability margin $$ m$$ of 0.05. For asymmetric focusing, we set $$ {\gamma }_{+}$$ to 1 and $$ {\gamma }_{-}$$ to 0, as recommended by ASL.

### Evaluation metrics

In this study, we regarded PPI prediction as a multi-category classification task. The dataset used in our study had an imbalanced distribution of samples across different categories. To effectively evaluate the model’s performance on this imbalanced dataset, we employed F1-measure as the evaluation metric. F1-measure is a widely-used evaluation metric for imbalanced datasets because it takes into account both precision and recall, providing a balanced measure of model performance [53].

$$ precision=\frac{TP}{TP+FP}$$

$$ recall=\frac{TP}{TP+FN}$$

$$ F1-measure=\frac{2*precision*recall}{precision+recall}$$

Among them, TP, FP, TN, and FN represent the number of predicted true positive, false positive, true negative, and false negative samples, respectively.

## Results and discussion

### Multimodal pre-training features offer a better representation of protein

The predictive performance of a model is directly influenced by the quality of its input features. In this study, we aimed to select high-quality features for protein vertices in the PPI network. To achieve this, we compared the sequence features of proteins with features obtained from the latest multimodal pre-training model of proteins. Experimental results presented in Table 2 exhibited that multimodal pre-training features can more effectively represent proteins. The sequence features used in our study were derived from the GNN-PPI method, a classic approach for predicting PPI. These sequence features consisted of 13 dimensions, with the first 5 dimensions capturing the co-occurrence similarity features of amino acids, and the remaining 8 dimensions representing the similarity features of electrostatic and hydrophobic interactions between amino acids. Through a combination of one layer of CNN and linear transformation, these 13-dimensional sequence features were transformed into 512-dimensional sequence features, which were utilized in our study. For the multimodal pre-training features, we employed the latest and more refined pre-training model called MASSA. This model leverages protein data from various modalities including protein sequences, structures, gene ontology annotations, motifs, and region positions to extract comprehensive protein features at a higher level. Subsequently, we constructed a GIN model and used the protein sequence features as well as the multimodal pre-training features as vertex features in the PPI network graph to predict PPI.

In the case of the SHS27K dataset, the GIN model based on multimodal pre-trained features exhibited the improved performance under three different dataset partitioning algorithms (Random, BFS, and DFS). Specifically, the F1-measure increased by approximately 0.7%, 7%, and 4%, respectively. As for the SHS148K dataset, the GIN network based on multimodal pre-trained features performed improvements of 1%, 4%, and 1% under the Random, BFS, and DFS algorithms, respectively. These experimental findings indicated that when using graph neural networks for predicting PPI, multimodal pre-trained features offered a better representation of protein compared to sequence features alone.

**Table 2:** Performance comparison between multimodal pre-training features and sequence features

Feature	SHS27k			SHS148K		
	Random (%)	BFS (%)	DFS (%)	Random (%)	BFS (%)	DFS (%)
Sequence features	88.21$\pm$0.47	63.26$\pm$3.70	74.31$\pm$3.44	91.98$\pm$0.20	65.50$\pm$4.59	82.24$\pm$0.73
MASSA	88.99$\pm$0.80	70.34$\pm$0.67	78.32$\pm$1.60	92.52$\pm$0.17	69.99$\pm$4.60	83.19$\pm$1.26

### Local subgraph features can enhance the performance of the model

In this study, we proposed a novel operation for updating vertex information in GIN. Instead of directly aggregating information from neighboring vertices in a star operation, we updated the vertex information through the features of the vertex’s subgraph. To assess the contribution of local subgraph features to the model’s performance, we conducted ablation experiments based on global graph features, local subgraph features, and their combined features. The global graph features were extracted directly using the GIN model, while the local subgraph features were extracted using the GIN-AK. The combined features were obtained by fusing global graph features and local subgraph features. Experimental results presented in Table 3 indicated that, based solely on global graph features and local subgraph features, the latter performed approximately 5% better than the former for the BFS partitioning algorithm on both SHS27K and SHS148K datasets, while they exhibited similar performance under the other two partitioning algorithms. However, after combining global graph features and local subgraph features, the combined model showed better performance than the global graph features-based and local subgraph features-based models under the two partitioning algorithms (Random and DFS) of the SHS148K dataset and all partitioning algorithms of the SHS27K dataset. Although the F1-measure of the combined model was 1% lower than that of the local subgraph features-based model under the BFS partitioning algorithm of the SHS148K dataset, these findings still showed that incorporating local subgraph features on the basis of global graph features could enhance the model’s performance.

**Table 3:** Performance comparison of global graph, subgraph, and their combined features

Feature	SHS27k			SHS148K		
	Random (%)	BFS (%)	DFS (%)	Random (%)	BFS (%)	DFS (%)
Subgraph	89.15$\pm$0.69	74.08$\pm$0.32	77.76$\pm$2.55	90.84$\pm$0.32	75.97$\pm$3.64	82.87$\pm$0.52
Global Graph	88.99$\pm$0.80	70.34$\pm$0.67	78.32$\pm$1.60	92.52$\pm$0.17	69.99$\pm$4.60	83.19$\pm$1.26
Combination	90.04$\pm$0.55	76.08$\pm$1.22	79.67$\pm$4.05	92.76$\pm$0.17	74.62$\pm$4.35	84.47$\pm$1.20

### Selection of parameter *k* in *k*-hop

To select the most effective parameter *k* in *k*-hop, we conducted experiments on the SHS27K dataset using both *k* values of 1 and 2. Experiment results, as presented in Table 4, showed that a *k* value of 1 outperformed the alternative. Due to the significant computational cost associated with calculating subgraphs for all vertices, we decided against conducting further experiments on the SHS27K dataset where the *k* value was greater than or equal to 3. Additionally, given the large size of the SHS148K dataset, it was not feasible to perform subgraph calculation experiments with a *k* value greater than or equal to 2 on our device. As a result, we ultimately settled on a *k* value of 1.

**Table 4:** Performance comparison of different *k* values based on SHS27K

k-hop	SHS27K		
	Random (%)	BFS (%)	DFS (%)
1-hop	90.23$\pm$0.31	78.51$\pm$5.25	79.81$\pm$3.43
2-hop	90.25$\pm$0.25	68.29$\pm$5.58	76.18$\pm$1.19

### The impact of ASL on the performance of the model

In the previous experiments, we utilized the BCE loss function, which is a type of symmetric loss function that does not address the issue of imbalanced sample sizes with different interaction categories in the dataset. To address this limitation, we introduced the ASL and conducted some experiments using it. Experimental results presented in Table 5 revealed that, under the three partitioning algorithms (Random, BFS, and DFS) on both SHS27K and SHS148K datasets, the model based on ASL achieved higher F1-measure than the model based on the BCE loss function. Specifically, in the SHS27K dataset, F1-measure increased by approximately 0.20%, 2%, and 0.15%, respectively, while in the SHS148K dataset, F1-measure increased by approximately 1%, 1%, and 2%, respectively. These results illustrated that utilizing the ASL can effectively enhance the performance of the model on imbalanced datasets.

**Table 5:** Performance comparison of asymmetric (ASL) and symmetric (BCE) loss functions

Loss Function	SHS27k			SHS148K		
	Random (%)	BFS (%)	DFS (%)	Random (%)	BFS (%)	DFS (%)
BCE	90.04$\pm$0.55	76.08$\pm$1.22	79.67$\pm$4.05	92.76$\pm$0.17	74.62$\pm$4.35	84.47$\pm$1.20
ASL	90.23$\pm$0.31	78.51$\pm$5.25	79.81$\pm$3.43	93.34$\pm$0.12	75.14$\pm$2.63	86.07$\pm$1.05

### Performance comparison of GNNGL-PPI with the state-of-the-art methods

In this study, GNNGL-PPI exhibited good performance across three different partitioning algorithms on the SHS27K and SHS148K datasets. To comprehensively assess its performance, we conducted a comparison with two machine learning methods, including Random Forest (RF) and Logistic Regression (LR), as well as four deep learning methods like PIPR, GNN-PPI, LDMGNN [54], and SemiGNN-PPI. Experimental results (Table 6) showed that GNNGL-PPI consistently outperformed these state-of-the-art methods in terms of F1-measure under all three partitioning algorithms on both datasets. These findings affirmed the reliability of GNNGL-PPI as a PPI predictor.

**Table 6:** Performance comparison of GNNGL-PPI with the state-of-the-art methods

Method	SHS27k			SHS148K		
	Random (%)	BFS (%)	DFS (%)	Random (%)	BFS (%)	DFS (%)
RF	78.45$\pm$0.08	37.67$\pm$1.57	35.55$\pm$2.22	82.10$\pm$0.20	38.96$\pm$1.94	43.26$\pm$3.43
LR	71.55$\pm$0.93	43.06$\pm$5.05	48.51$\pm$1.87	67.00$\pm$0.07	47.45$\pm$1.42	51.09$\pm$2.09
PIPR	83.31$\pm$0.75	44.48$\pm$4.44	57.80$\pm$3.24	90.05$\pm$2.59	61.83$\pm$10.23	63.98$\pm$0.76
GNN-PPI	87.91$\pm$0.39	63.81$\pm$1.79	74.72$\pm$5.26	92.26$\pm$0.10	71.37$\pm$5.33	82.67$\pm$0.85
LDMGNN	89.34$\pm$0.44	74.56$\pm$3.03	78.20$\pm$2.69	92.38$\pm$0.08	73.98$\pm$5.51	83.79$\pm$0.95
SemiGNN-PPI	89.51$\pm$0.46	72.15$\pm$2.87	78.32$\pm$3.15	92.40$\pm$0.22	71.78$\pm$3.56	85.45$\pm$1.17
GNNGL-PPI	90.23$\pm$0.31	78.51$\pm$5.25	79.81$\pm$3.43	93.34$\pm$0.12	75.14$\pm$2.63	86.07$\pm$1.05

### Performance comparison between GNNGL-PPI and state-of-the-art methods under the multi-classification evaluation metrics

In addition to using binary classification evaluation metrics, we further utilized multi-classification evaluation metrics such as Weighted precision, Weighted recall, Weighted f1-score, Macro precision, Macro recall, Macro f1-score, Micro precision, Micro recall, and Micro f1-score to evaluate the model’s performance. Experimental results (Table 7) showed that GNNGL-PPI exhibited superior performance compared to other state-of-the-art methods. These outcomes highlighted the effectiveness of GNNGL-PPI in predicting the multi-category of PPI.

**Table 7:** Performance comparison between GNNGL-PPI and other state-of-the-art methods under the multi-classification evaluation metrics

Method	Multi-classification evaluation metrics	SHS27K			SHS148K		
		Random (%)	BFS (%)	DFS (%)	Random (%)	BFS (%)	DFS (%)
GNNGL-PPI	Weighted precision	89.49$\pm$0.43	76.41$\pm$1.14	76.49$\pm$2.22	92.96$\pm$0.20	75.37$\pm$2.71	84.85$\pm$0.65
LDMGNN		89.13$\pm$0.55	73.14$\pm$2.32	76.11$\pm$0.08	92.89$\pm$0.33	72.56$\pm$3.32	83.35$\pm$1.35
GNN-PPI		88.97$\pm$0.17	69.75$\pm$9.24	70.92$\pm$1.94	92.87$\pm$0.07	70.91$\pm$2.55	83.08$\pm$0.56
GNNGL-PPI	Weighted recall	90.81$\pm$0.43	79.38$\pm$2.02	82.92$\pm$2.98	94.00$\pm$0.07	78.88$\pm$6.02	88.74$\pm$0.38
LDMGNN		88.41$\pm$0.22	74.06$\pm$3.47	81.15$\pm$2.79	92.04$\pm$0.14	65.23$\pm$2.76	80.88$\pm$1.67
GNN-PPI		88.10$\pm$0.32	71.68$\pm$9.55	73.67$\pm$2.40	91.63$\pm$0.25	59.12$\pm$6.67	82.60$\pm$1.01
GNNGL-PPI	Weighted f1-score	90.13$\pm$0.41	77.42$\pm$0.86	79.39$\pm$2.57	93.47$\pm$0.07	76.66$\pm$4.37	86.69$\pm$0.60
LDMGNN		88.73$\pm$0.39	72.81$\pm$2.39	78.16$\pm$1.30	92.45$\pm$0.20	67.32$\pm$2.71	81.86$\pm$1.04
GNN-PPI		88.50$\pm$0.25	68.30$\pm$1.91	71.87$\pm$1.64	92.24$\pm$0.10	62.53$\pm$4.21	82.72$\pm$0.77
GNNGL-PPI	Macro precision	86.36$\pm$0.66	70.00$\pm$5.68	72.63$\pm$2.65	89.76$\pm$0.47	72.83$\pm$2.70	78.44$\pm$0.77
LDMGNN		85.92$\pm$0.40	70.08$\pm$2.81	73.16$\pm$0.47	89.60$\pm$0.30	71.60$\pm$3.26	78.26$\pm$0.87
GNN-PPI		85.88$\pm$0.40	57.88$\pm$20.34	67.37$\pm$1.51	89.64$\pm$0.15	69.18$\pm$3.32	77.65$\pm$0.57
GNNGL-PPI	Macro recall	86.52$\pm$0.60	72.22$\pm$4.15	78.09$\pm$2.74	90.20$\pm$0.06	75.55$\pm$5.97	83.71$\pm$0.31
LDMGNN		83.89$\pm$0.67	64.32$\pm$2.12	74.34$\pm$1.63	87.32$\pm$0.07	58.13$\pm$1.70	74.14$\pm$1.84
GNN-PPI		83.43$\pm$0.45	52.88$\pm$8.32	69.44$\pm$1.37	87.07$\pm$0.40	54.24$\pm$8.07	72.37$\pm$0.88
GNNGL-PPI	Macro f1-score	86.40$\pm$0.33	70.37$\pm$4.35	74.99$\pm$2.72	89.97$\pm$0.26	73.70$\pm$3.83	80.83$\pm$0.55
LDMGNN		84.80$\pm$0.52	65.85$\pm$2.10	72.80$\pm$0.35	88.47$\pm$0.16	62.11$\pm$3.11	75.77$\pm$1.47
GNN-PPI		84.55$\pm$0.13	52.96$\pm$14.06	68.00$\pm$1.34	88.30$\pm$0.18	58.62$\pm$5.55	73.82$\pm$0.66
GNNGL-PPI	Micro precision	89.49$\pm$0.49	75.74$\pm$1.09	76.23$\pm$2.14	92.95$\pm$0.23	74.77$\pm$3.26	84.35$\pm$0.77
LDMGNN		89.24$\pm$0.48	73.52$\pm$2.64	76.10$\pm$0.18	93.05$\pm$0.32	72.28$\pm$3.12	82.90$\pm$1.39
GNN-PPI		89.11$\pm$0.25	71.54$\pm$5.70	70.44$\pm$2.11	92.98$\pm$0.09	70.03$\pm$1.74	83.27$\pm$0.57
GNNGL-PPI	Micro recall	90.82$\pm$0.43	79.39$\pm$2.03	82.93$\pm$2.98	94.01$\pm$0.07	78.88$\pm$6.03	88.76$\pm$0.36
LDMGNN		88.42$\pm$0.22	74.06$\pm$3.47	81.15$\pm$2.79	92.05$\pm$0.15	65.23$\pm$2.77	80.88$\pm$1.67
GNN-PPI		88.11$\pm$0.32	71.69$\pm$9.55	73.34$\pm$2.26	91.64$\pm$0.25	59.12$\pm$6.68	82.60$\pm$1.02
GNNGL-PPI	Micro f1-score	90.15$\pm$0.39	77.49$\pm$0.56	79.43$\pm$2.53	93.48$\pm$0.09	76.72$\pm$4.29	86.50$\pm$0.56
LDMGNN		88.82$\pm$0.32	73.74$\pm$2.48	78.12$\pm$1.10	92.54$\pm$0.20	68.52$\pm$2.26	81.86$\pm$0.94
GNN-PPI		88.60$\pm$0.29	70.81$\pm$1.72	71.83$\pm$1.61	92.31$\pm$0.09	63.86$\pm$3.43	82.87$\pm$0.70

### Performance comparison of some statistical tests between GNNGL-PPI and state-of-the-art methods

In this study, we dealt with an imbalanced dataset where the number of samples across different categories varied. To accurately evaluate the model’s performance on this imbalanced dataset, we opted for the F1 metric as it provided a more accurate reflection of the model’s true performance. Consequently, we conducted some statistical tests on the F1 metric of three methods including GNNPPI, LDMGN, and GNNGL-PPI using three distinct partitioning algorithms on both SHS27K and SHS148K datasets. Experiment results, as shown in Table 8, indicated that GNNGL-PPI exhibited slightly superior performance across two statistical testing metrics compared to two other state-of-the-art methods.

**Table 8:** Performance comparison of some statistical tests between GNNGL-PPI and state-of-the-art methods

Rank		Number of cases		Rank Mean	Sum of Ranks	
GNNPPI-GNNGLPPI	Negative Rank	18^a^		9.50	171.00	
	Positive Rank	0^b^		0.00	0.00	
	Bind Value	0^c^				
	Total	18				
LDMGN-GNNGLPPI	Negative Rank	17^d^		9.53	162.00	
	Positive Rank	1^e^		9.00	9.00	
	Bind Value	0^f^				
	Total	18				
(a) GNNPPI < GNNGLPPI (b) GNNPPI > GNNGLPPI (c) GNNPPI = GNNGLPPI (d) LDMGN < GNNGLPPI (e) LDMGN > GNNGLPPI (f) LDMGN = GNNGLPPI						
Statistic tests (a)	Z		Progressive significance (two tailed)			p-value
GNNPPI-GNNGLPPI	-3.724^b^		0.000			0.000196
LDMGN-GNNGLPPI	-3.332^b^		0.001			0.000863
(a) Wilconxon sign rank test (b) Based on positive rank						

### Statistical analysis of prediction accuracy of GNNGL-PPI and GNN-PPI under different interaction categories

**Fig. 2:**
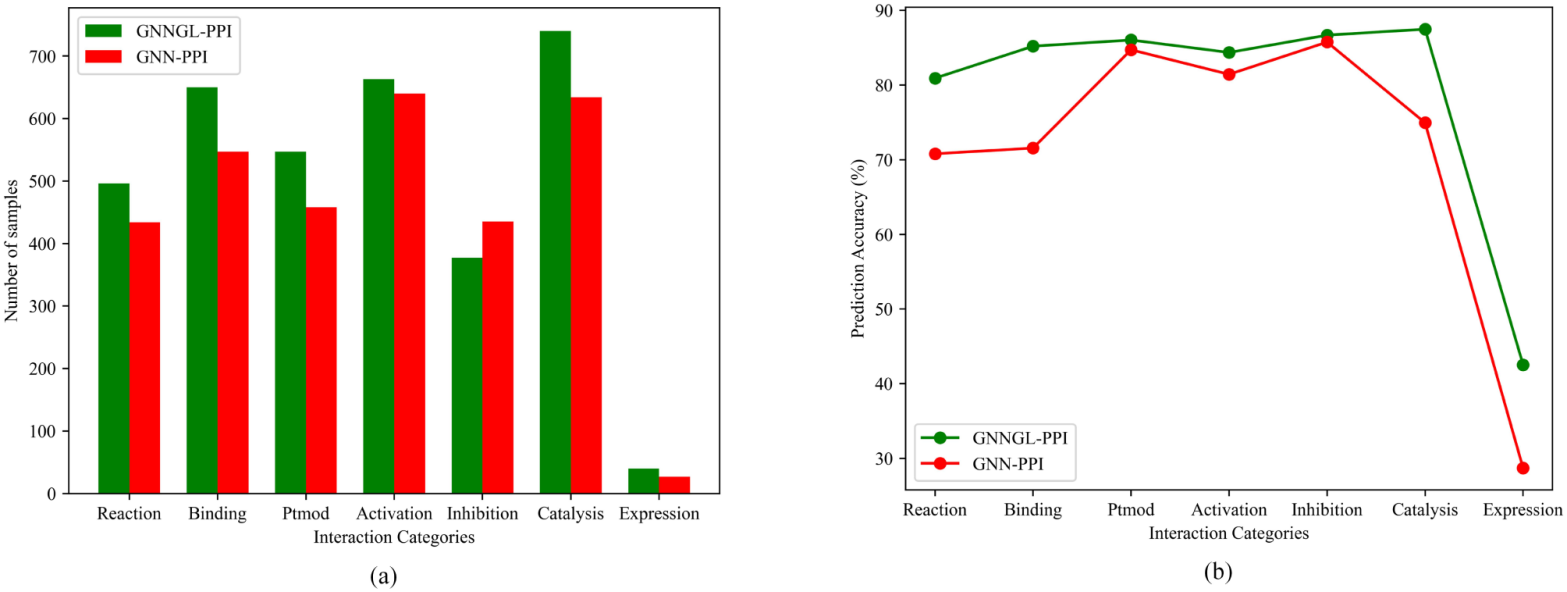
Performance comparison between GNNGL-PPI and GNN-PPI across different interaction categories using the BFS partitioning algorithm on the SHS27K dataset. (**a**) In each interaction category, GNNGL-PPI outperformed GNN-PPI by predicting more correct samples (green color) as compared to the latter (red color). Particularly in the Expression category, GNNGL-PPI correctly predicted 40 positive samples, while GNN-PPI only achieved 27. (**b**) With the exception of the Action and Inhibition interaction categories, the prediction accuracy of both methods was similar. However, in the remaining five interaction categories, GNNGL-PPI exhibited a prediction accuracy that was more than 10% higher than that of GNN-PPI.

Table 6 showed that the GNNGL-PPI method outperformed other state-of-the-art methods for comparison under three different partitioning algorithms on both the SHS27K and SHS148K datasets. To further evaluate the predictive performance of GNNGL-PPI across different interaction categories, we compared its prediction accuracy with the GNN-PPI method under the BFS partitioning algorithm using the SHS27K dataset (Fig. 2). Statistical analysis revealed that for the interaction category Reaction with 3164 samples (Table 1), after being divided by the BFS algorithm, 613 test samples were obtained. GNNGL-PPI correctly predicted 496 of the 569 samples it identified, yielding an accuracy of 80.91% (Fig. 2a). In comparison, GNN-PPI correctly predicted 434 of the 510 samples, achieving an accuracy of 70.79%. Similarly, for the Binding, Ptmod, Activation, Inhibition, Catalysis, and Expression categories, GNNGL-PPI achieved a higher accuracy (within parentheses) than that of GNN-PPI (85.19%, 71.55%), (86.02%, 71.55%), (84.35%, 81.42%), (86.67%, 85.74%), (87.47%, 74.94%), and (42.55%, 28.72%), respectively (Fig. 2b). In all interaction categories except for Action and Inhibition, GNNGL-PPI exhibited classification accuracy that was more than 10% higher than that of GNN-PPI. Notably, under the Expression category, there were only 94 test samples. Despite this, GNNGL-PPI and GNN-PPI correctly predicted 27 and 40 samples, respectively, with an accuracy improvement of 13.83%. This suggested that our proposed GNNGL-PPI method could learn features from small sample sizes and made accurate predictions, mitigating the problem of low prediction accuracy due to imbalanced dataset categories with small sample sizes.

### Interpretability analysis of the effectiveness of GNNGL-PPI

**Fig. 3:**
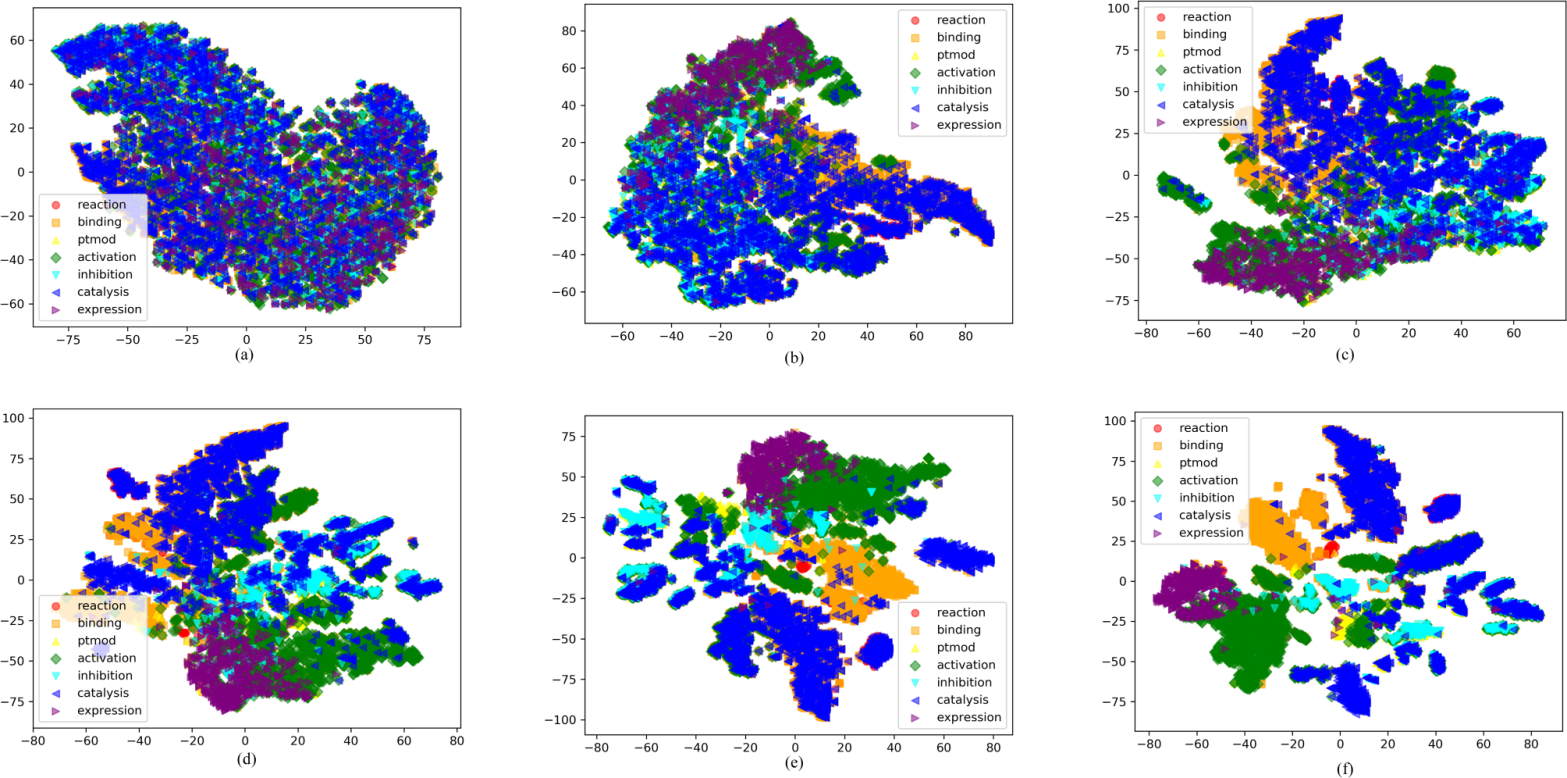
Interpretability analysis of the effectiveness of GNNGL-PPI. We used t-SNE algorithm to elucidate GNNGL-PPI’s effectiveness by contrasting the clustered visualizations resulting from dimensionality reduction for multiple different epochs of the model training of GNNGL-PPI. The clustering visualization results, represented as (**a**) to (**f**), corresponded to the features learned in the first, fifth, 20th, 50th, 100th, and 200th epochs of model training, respectively. Upon examining these results, a clear pattern emerged: data points representing different interaction categories gradually moved apart as the number of training epochs increased, while data points representing the same interaction category gradually clustered together. The clustering visualization results showed that GNNGL-PPI was an effective PPI multi-category predictor

To thoroughly evaluate the effectiveness of GNNGL-PPI, we applied the widely recognized dimensionality reduction algorithm t-SNE [55] to the SHS27K dataset. This dataset was partitioned using the Random alogrithm. t-SNE proved to be an ideal choice for our analysis as it preserved the proximity of closely positioned data points even after reducing their dimensions. At the same time, it effectively maintained the separation between originally distant data points. We utilized t-SNE to gain insights into the effectiveness of GNNGL-PPI by visualizing the clustered representations resulting from dimensionality reduction at different epochs of model training. Due to the rapid convergence of GNNGL-PPI in the early stages of training, we applied t-SNE to reduce the dimensionality of features learned in the 1st, 5th, 20th, 50th, 100th, and 200th epochs. The clustering visualization obtained after one epoch of model training (Fig. 3a) revealed that data points representing different interaction categories were intertwined, with no distinct separation between them. However, as the number of training epochs increased, the clustering visualizations after the 5th, 20th, 50th, and 100th epochs (Fig. 3b to e) clearly showed that data points representing different interaction categories gradually moved away from each other, while data points representing the same interaction category started to cluster together. By the 200th epoch, clear boundaries between data points of different interaction categories were observed (Fig. 3f). It’s important to note that since this study treated PPI as a multi-category classification task, some data points from different interaction categories may still appear in the same cluster. Nevertheless, the clustering visualization results from different training epochs indicated that GNNGL-PPI was an effective method for predicting multi-category of PPI.

## Discussions

Although GNNGL-PPI has exhibited a fine performance in predicting multiple categories of protein-protein interactions and can provide explanatory analysis for its effectiveness, it also has certain shortcomings:

1. The training of GNNGL-PPI was conducted on two publicly available benchmark datasets, and the model’s learned deep features were limited. This limitation may result in a gap between the generalization performance of GNNGL-PPI and users’ expected thresholds.
2. GNNGL-PPI relied on sequence features and GO annotations of proteins, while overlooking protein structural features. However, protein structural features often play a crucial role in the performance of PPI models.
3. The utilization of GNNGL-PPI requires users to input protein-protein interaction networks, which undoubtedly increases the complexity for non-professionals and restricts the application and promotion of GNNGL-PPI.

In response to these shortcomings, we have carefully considered the issues and proposed potential solutions:

1. To address the limited deep features learned by GNNGL-PPI, we suggest crawling a more comprehensive PPI interaction dataset from databases. By constructing a more extensive PPI interaction network for training GNNGL-PPI, we can gradually enhance its generalization performance.
2. In order to incorporate protein structural features into GNNGL-PPI, we recommend extracting such features from protein topology graphs or three-dimensional grids using graph neural networks or 3D-CNN. This approach will enrich the protein features and improve the model’s performance.
3. To alleviate the challenges faced by non-professionals in utilizing GNNGL-PPI, we propose developing an online service tool. With this tool, users would only need to input protein pairs, and the tool would automatically construct a protein-protein interaction network and predict the interaction categories between proteins.

In order to further improve the performance of GNNGL-PPI, we believe that in-depth research should be conducted in the following areas in the future:

- Introducing self-supervised contrastive learning into the GNNGL-PPI method enhances its feature extraction ability, enabling the method to learn more key implicit features that affect multi-category prediction of protein-protein interactions.
- At present, the protein features used are still relatively single, and multiple modal information such as protein sequences and structures should be integrated to promote the model to achieve better performance based on the rich features of proteins.
- Deepening the interpretive analysis of the effectiveness of protein-protein interaction prediction methods will not only promote the application of this method, but also provide support for the research of new methods.

GNNGL-PPI not only provides PPI prediction services to support users in understanding the mechanism of protein-protein interaction, but also the predicted PPI categories or PPI networks constructed from them can provide deep and rich features for drug-target interaction prediction and other related tasks, which is helpful for drug screening, repositioning, and target recognition research. We will briefly provide relevant work in Appendix A.

## Conclusions

GNNGL-PPI is a novel method for predicting multi-category of PPI based on graph neural networks. This innovative approach leverages the power of GIN and GIN-AK to extract the features from both the global graph and local subgraph of the PPI network. Additionally, the use of ASL helps enhance the model’s performance on imbalanced datasets. Experimental results have showed that GNNGL-PPI surpasses the performance of existing state-of-the-art methods for PPI prediction on two standard datasets. The effectiveness of GNNGL-PPI is further supported by the t-SNE algorithm, which provides visual evidence of the model’s capability. Therefore, GNNGL-PPI is a reliable multi-category prediction tool for protein-protein interactions.

## Appendix A

Here, we will briefly mention that predicting protein-protein interactions can significantly contribute to drug-target interactions, target identification, and other related research in drug discovery, ultimately advancing drug development.

TripletMultiDTI [56] utilized protein-protein interaction (PPI) and drug-drug interaction (DDI) networks as supplementary knowledge. It integrated multimodal information, including drugs, proteins, DDI networks, and PPI networks, as inputs to predict the affinity of drug-target pairs. Furthermore, a review article [57] elaborated on the crucial role of protein-protein interactions in performing essential cellular functions. These interactions have been pivotal drug targets for the past two decades and are fundamental to drug development and design. Additionally, reference [58] provided a detailed exploration of the vital role of protein-protein interactions in structural biology and drug discovery.

## Data Availability

The source data and code repository can be accessed at https://github.com/dldxzx/GNNGL-PPI.
